# Chicago College of Dental Surgery

**Published:** 1886-05

**Authors:** 


### CHICAGO COLLEGE OF DENTAL SURGERY.

### The Address of Dr. James A. Swasey, President of the Board of Directors, to the Graduating Class.

The presentation of your diplomas is the last official act of your Alma Mater. We have placed you in the front rank of dental graduates, for we have required of you higher preliminary qualifications, a more rigid final examination, and a longer College course than is required in most similar institutions. If you would honor the profession you have chosen, there are many things you must do, and some things you must not do.

First, you must continue you studies by frequent reference to the notes you have taken of the valuable lectures given by your professors, and by your numerous text-books. But rely not upon these alone. Procure the best literature of the profession, and read it carefully. Join all dental societies that it will be possible for you to attend. And note well the experience of your seniors, for what they have acquired by perseverance and study, should be of service to you and your patients. Do not think you can accomplish with little effort what others have worked a lifetime to acquire. Study carefully all cases, to make a correct diagnosis. This is not always an easy thing to do. In giving your advice be honest, and in every operation that you perform do your best, that your patients may not have occasion to feel that they have been misled by your advice, or have suffered from your lack of careful attention. The successful dentists are those who are thorough in every operation, however insignificant. Emulate them, and you will rise in the race for fame. These are some of the things you should do. Now the things you should not do.

Do not compromise your personal dignity by being too familiar.

Do not think your patrons come to you to be amused, for there is very little that is amusing in a well regulated dental office.

Do not boast of your superior skill; your patients will soon discover for themselves whether you have any or not.

Do not have the only set of instruments of the kind ever manufactured, or the only panacea for any pain.

Do not imagine that every young lady who consults you is hopelessly in love with you, and dying for an acquaintance.

Do not go to church for the sake of getting practice, but for a higher and nobler object. If you must resort to this or starve, take a pew in but one church at a time.

Do not complain of being overworked and rushed with appointments, when you are eagerly waiting and watching for patients, and your landlady is as eagerly waiting and watching for her board bill.

Do not imagine that you have a great reputation after a few months' practice. It takes many years to make a reputation in dentistry, and a lifetime to keep it.

Do not forget that your Alma Mater will watch anxiously for your future; that she will be proud of your successes and deplore your failures; that any needed advice will always be most cheerfully given, and that she hopes that her festive occasions will always be cheered by your presence.

